# Reliable Quantification of the Potential for Equations Based on Spot Urine Samples to Estimate Population Salt Intake: Protocol for a Systematic Review and Meta-Analysis

**DOI:** 10.2196/resprot.6282

**Published:** 2016-09-21

**Authors:** Liping Huang, Michelle Crino, Jason HY Wu, Mark Woodward, Mary-Anne Land, Rachael McLean, Jacqui Webster, Batsaikhan Enkhtungalag, Caryl A Nowson, Paul Elliott, Mary Cogswell, Ulla Toft, Jose G Mill, Tania W Furlanetto, Jasminka Z Ilich, Yet Hoi Hong, Damian Cohall, Leonella Luzardo, Oscar Noboa, Ellen Holm, Alexander L Gerbes, Bahaa Senousy, Sonat Pinar Kara, Lizzy M Brewster, Hirotsugu Ueshima, Srinivas Subramanian, Boon Wee Teo, Norrina Allen, Sohel Reza Choudhury, Jorge Polonia, Yoshinari Yasuda, Norm RC Campbell, Bruce Neal, Kristina S Petersen

**Affiliations:** ^1^ The George Institute for Global Health Peking University Health Science Center Beijing China; ^2^ The George Institute for Global Health The University of Sydney Sydney Australia; ^3^ The George Institute for Global Health University of Oxford Oxford United Kingdom; ^4^ Department of Epidemiology Johns Hopkins University Baltimore, MD United States; ^5^ Departments of Preventive and Social Medicine/Human Nutrition University of Otago Dunedin New Zealand; ^6^ Nutrition Research Center, Public Health Institute Ulaanbaatar Mongolia; ^7^ Deakin University, Institute for Physical Activity and Nutrition Research Geelong Australia; ^8^ Department of Epidemiology and Biostatistics and MRC-PHE Centre for Environment and Health School of Public Health Imperial College London London United Kingdom; ^9^ Division of Heart Disease and Stroke Prevention National Center for Chronic Disease Prevention and Health Promotion, Centers for Disease Control and Prevention Atlanta, GA United States; ^10^ Research Centre for Prevention and Health Glostrup University Hospital Glostrup Denmark; ^11^ Center of Health Sciences Department of Physiological Sciences Federal University of Espírito Santo Vitória Brazil; ^12^ Postgraduation Program in Medicine Medical Sciences-Universidade Federal do Rio Grande do Sul Porto Alegre Brazil; ^13^ Nutrition, Food and Exercise Sciences Florida State University Tallahassee, FL United States; ^14^ Department of Physiology Faculty of Medicine University of Malaya Kuala Lumpur Malaysia; ^15^ Faculty of Medical Sciences The University of the West Indies Cave Hill Barbados; ^16^ Centro de Nefrología, Departamento de Fisiopatología Hospital de Clínicas Facultad de Medicina Universidad de la República Montevideo Uruguay; ^17^ Centro de Nefrología Hospital de Clínicas, Facultad de Medicina, Universidad de la Republica Montevideo Uruguay; ^18^ Medical Department Nykøbing Falster Hospital Nykøbing Falster Denmark; ^19^ Liver Centre Munich Department of Medicine II Hospital of the University of Munich, KUM Munich Germany; ^20^ Department of Gastroenterology and Hepatology Ain Shams University School of Medicine Cairo Egypt; ^21^ Namik Kemal University, Faculty of Medicine, Department of Internal Medicine Tekirdag Turkey; ^22^ Departments of Internal and Vascular Medicine, Academic Medical Center Amsterdam Netherlands; ^23^ Department of Public Health Shiga University of Medical Science Tsukinowa-cho Seta, Otsu Shiga Japan; ^24^ Department of Medicine, Division of Nephrology Yong Loo Lin School of Medicine National University of Singapore Singapore; ^25^ Northwestern University Chicago, IL United States; ^26^ Department of Epidemiology and Research National Heart Foundation Hospital & Research Institute Dhaka Bangladesh; ^27^ Faculdade Medicina da Universidade do Porto, Porto, Portugal & Unidade Hipertensão ULS Matosinhos Portugal; ^28^ Department of CKD Initiatives/Nephrology, Nagoya University Graduate School of Medicine Nagoya Japan; ^29^ Departments of Medicine, Physiology and Pharmacology and Community Health Sciences, O’Brien Institute for Public Health Libin Cardiovascular Institute of Alberta University of Calgary Calgary, AB Canada; ^30^ Royal Prince Alfred Hospital Sydney Australia

**Keywords:** dietary salt, sodium, spot urine collection, 24-hour urine collection, meta-analysis, systematic review

## Abstract

**Background:**

Methods based on spot urine samples (a single sample at one time-point) have been identified as a possible alternative approach to 24-hour urine samples for determining mean population salt intake.

**Objective:**

The aim of this study is to identify a reliable method for estimating mean population salt intake from spot urine samples. This will be done by comparing the performance of existing equations against one other and against estimates derived from 24-hour urine samples. The effects of factors such as ethnicity, sex, age, body mass index, antihypertensive drug use, health status, and timing of spot urine collection will be explored. The capacity of spot urine samples to measure change in salt intake over time will also be determined. Finally, we aim to develop a novel equation (or equations) that performs better than existing equations to estimate mean population salt intake.

**Methods:**

A systematic review and meta-analysis of individual participant data will be conducted. A search has been conducted to identify human studies that report salt (or sodium) excretion based upon 24-hour urine samples and spot urine samples. There were no restrictions on language, study sample size, or characteristics of the study population. MEDLINE via OvidSP (1946-present), Premedline via OvidSP, EMBASE, Global Health via OvidSP (1910-present), and the Cochrane Library were searched, and two reviewers identified eligible studies. The authors of these studies will be invited to contribute data according to a standard format. Individual participant records will be compiled and a series of analyses will be completed to: (1) compare existing equations for estimating 24-hour salt intake from spot urine samples with 24-hour urine samples, and assess the degree of bias according to key demographic and clinical characteristics; (2) assess the reliability of using spot urine samples to measure population changes in salt intake overtime; and (3) develop a novel equation that performs better than existing equations to estimate mean population salt intake.

**Results:**

The search strategy identified 538 records; 100 records were obtained for review in full text and 73 have been confirmed as eligible. In addition, 68 abstracts were identified, some of which may contain data eligible for inclusion. Individual participant data will be requested from the authors of eligible studies.

**Conclusions:**

Many equations for estimating salt intake from spot urine samples have been developed and validated, although most have been studied in very specific settings. This meta-analysis of individual participant data will enable a much broader understanding of the capacity for spot urine samples to estimate population salt intake.

## Introduction

Excess consumption of salt causes high blood pressure [1-3], which is a leading risk factor for premature death worldwide [4]. The World Health Organization (WHO) recommends a maximum daily salt intake for individuals and populations of 5g/person/day [5]. Most countries (in which salt intake has been measured) exceed this recommendation, often by a large margin [6-8]. In response to the United Nations’ high-level meeting on the prevention and control of non-communicable diseases in 2011, the WHO has set a target for Member States to reduce population salt consumption by 30% by 2025 [9].

To document a 30% reduction in average population salt consumption, knowledge of baseline and follow-up intake levels are required. The accepted method for measuring average population salt intake is to collect 24-hour urine samples from a subset of the population [10]. However, 24-hour urine samples are onerous for survey participants and researchers to collect, and typically the response rates in surveys are low [11]. Incomplete urine collections also represent a challenge, and although several methods have been used to identify incomplete collections (such as the use of para-aminobenzoic acid), limitations still exist with these methods [12]. Questionnaire-based dietary surveys are another method of estimating salt consumption but are even more problematic. This is principally because no robust scalable method exists for estimating discretionary salt use, which can represent a large proportion of the dietary salt consumed in many countries [13]. Systematic misreporting of foods consumed is another major challenge to questionnaire-based approaches [14].

Estimation equations based upon spot urine samples may offer a practical alternative method for measuring average population salt consumption. This method is less burdensome for participants and response rates are markedly higher, ranging from 73-100% [11]. Individual-level correlation of urinary salt concentration in a spot sample with that of a 24-hour urinary salt is not always high [15-25] and some authors have used this observation to suggest that spot urine samples cannot be used as an alternative to 24-hour sampling [26-29]. The relatively low individual-level correlation of spot samples with 24-hour urinary salt excretion, along with the relatively large day-to-day variability of individuals’ salt intake, makes it likely that spot urine sampling is inadequate for individual-level assessment in epidemiological studies. However, the method has an established role in estimating 24-hour excretion of pesticides and chemicals in the US National Health and Nutrition Examination Survey [30,31]. A strong likelihood exists that the mean salt concentration of spot urine samples for a given population is related to the mean 24-hour excretion of that same population.

A series of equations that estimate 24-hour urinary salt excretion from spot urine samples have been developed [32-36]. These equations have been derived in different populations, include a range of different covariates, and use spot urine samples voided at different times of the day. Some equations have been applied in populations external to those used for equation development, and different conclusions have been drawn about their validity [24,37-41]. It is possible that mean population 24-hour urinary salt excretion can be predicted from spot urine samples in some populations but not others, or that the different analytical approaches used have resulted in different findings. It is also possible that the small size of some studies has produced spurious findings.

We recently conducted a systematic review and meta-analysis of published aggregate data that included 10,414 participants from 35 countries, which showed that spot urine samples had excellent specificity (100%) and sensitivity (97%) at classifying mean population salt intake as above or below the WHO maximum target of 5g/day [42]. This result suggests that spot urine samples present a viable option for countries to determine salt intake levels and make objective decisions about the requirement for salt reduction strategies. Based on this meta-analysis, we identified for the first time a possible proportional bias, such that equations based on spot urine samples may overestimate consumption at lower levels and underestimate at higher levels of consumption. This proportional bias would be expected to result in an underestimation of the reduction in average salt intake if spot urine samples were used to monitor population salt consumption over time. However, due to this observation being based on a group-level meta-analysis, it should be interpreted with caution due to the possibility of confounding.

In many other areas of medical research, the combination of data at the individual participant level has enabled the resolution of comparable types of problems [43]. This is because analyses can be done in a standard way across all datasets, and because statistical precision is greatly increased by the larger volume of data available. In addition, bringing the many leaders working in this area together on a single project may synergize efforts to resolve the question at hand.

### Overall Goal and Specific Objectives

The overall goal of this initiative is to identify a reliable method for estimating mean population salt intake from spot urine samples. The method should provide an estimate that is directly comparable to measurements made using 24-hour urine samples with no evidence of proportional bias. The specific aims are:

To determine the direction and magnitude of the bias of existing equations for estimating mean population 24-hour salt excretion from spot urine samples compared to 24-hour urine samples, and how ethnicity, sex, age, body mass index (BMI), antihypertensive drug use, health status, and timing of spot urine collection may modify the performance of these equations.

To investigate if equations for estimating mean population 24-hour salt excretion from sequential spot urine samples can be used to estimate the mean change in population salt intake over time.

To develop a novel equation (or equations) that performs better than existing equations to estimate mean population salt intake and changes in mean population intake over time.

## Methods

This protocol involves a systematic review and meta-analysis of individual participant data. Based on the secondary analyses of existing data, separate regulatory reviews and ethics committee approvals will not be sought. The active participation of a lead investigator from every study will be encouraged, in order to maximize insight into the data included and obtain the broadest possible range of expertise available to address the research questions.

**Study inclusion and exclusion criteria.**

*Inclusion criteria:*

- Original research conducted in a human population
- Report salt (or sodium) excretion based upon 24-hour urine samples and spot urine samples in the same individuals

*Exclusion criteria:*

- Urinary salt (or sodium) not measured in both spot samples and 24-hour urine samples
- Urinary creatinine not measured in both spot samples and 24-hour urine samples
- Spot and 24-hour urine samples not collected in the same sample

**Table 1:** Variables to be collected and data format.

Variable		Format	
**Core variables required for the analyses**			
	1. Unique anonymous identifier		Numerical value
	2. Study identifier		Numerical value
	3. Country of participant recruitment		United States, United Kingdom, Japan, etc.
	4. Sex, female?		1=yes, 2=no
	5. Age		Numerical value (years)
	6. Height		Numerical value (cm)
	7. Weight		Numerical value (kg)
	8. Date of first 24-hour urine sample collection started		dd/mm/yyyy
	9. Start time of first 24-hour urine collection		hhmm
	10. End time of first 24-hour urine collection		hhmm
	11. 24-hour urine volume first sample		Numerical value (L)
	12. Sodium concentration in first 24-hour urine		Numerical value (mmol/L)
	13. Potassium concentration in first 24-hour urine		Numerical value (mmol/L)
	14. Creatinine concentration in first 24-hour urine		Numerical value (mmol/L)
	15. Date of first spot urine sample collection		dd/mm/yyyy
	16. Time of first spot urine sample collection		hhmm or 1=first morning, 2=morning, 3=afternoon, 4=evening, 5=random
	17. Was first spot sample part of 24-h collection?		1=yes, 2=no
	18. Sodium concentration in first spot urine		Numerical value (mmol/L)
	19. Potassium concentration in first spot urine		Numerical value (mmol/L)
	20. Creatinine concentration in first spot urine		Numerical value (mmol/L)
**Supplementary variables that should be included when available**			
	21. Race		1=White, 2=Black, 3=Asian, 4=other, 5=Hispanic
	22. Systolic Blood Pressure		Numerical value (mmHg)
	23. Diastolic Blood Pressure		Numerical value (mmHg)
	24. History of hypertension?		1=yes, 2=no, 3=unknown
	25. History of diabetes?		1=yes, 2=no, 3=unknown
	26. History of kidney disease?		1=yes, 2=no, 3=unknown
	27. History of heart disease or stroke?		1=yes, 2=no, 3=unknown
	28. Using any blood pressure lowering drug?		1=yes, 2=no, 3=unknown
	29. Using diuretic therapy?		1=yes, 2=no, 3=unknown
	30. Pregnancy status		1=yes, 2=no, 3=unknown
	31. Start time of first spot urine collection		hhmm
	32. End time of first spot urine collection		hhmm
	33. Volume of first spot urine collection		Numerical value (L)

When more than one spot urine sample has been collected from the same individual, items 8-20 will be sought for the additional time-points. To assess the capacity of spot urine samples to measure changes in sodium excretion over time (change analyses), a second set of spot and 24-hour urine sodium measures (collected 1 month or more after the first sample) will be sought for the second time-point (data items 8-20). Missing data will be recorded as blank.

### Study Inclusion and Exclusion Criteria

There will be no restrictions on language, study sample size, or characteristics of the study population. Inclusion and exclusion criteria are outlined in Textbox 1.

### Search Strategy

Search strategies were developed in consultation with a librarian at the University of Sydney, with the goal of identifying all studies that might contribute data. The electronic databases MEDLINE via OvidSP (1946-present), Premedline via OvidSP, EMBASE, Global Health via OvidSP (1910-present), and the Cochrane Library were searched using applicable terms (Multimedia Appendix 1). A keyword search using *24-hour urinary sodium excretion* was also conducted in the China National Knowledge Infrastructure. In addition, hand searches of the reference lists of eligible studies were completed, and academic colleagues working in the field were contacted to identify unpublished data. The same search will be repeated annually to identify and include new studies as they become available.

Two reviewers independently screened the titles and abstracts of all identified articles. All potentially relevant abstracts identified by either reviewer were obtained in full text, if available. The two reviewers screened the full text papers independently to determine eligibility, with disagreements settled by discussion between the two, or via consultation with a third author when necessary. Additionally, if there was doubt about whether an article contained data that could be used for this project, the article was retained and attempts will be made to contact the study’s authors. Abstracts (including conference proceedings) that were potentially eligible, but without full text available, were retained and attempts to contact the authors will be made. Non-English articles were found in the literature search, and in all cases the papers had an English abstract that was used to assess eligibility; authors will be contacted to determine eligibility for the project.

### Data Request

A standard set of data in a standardized format will be sought from each participating study (Table 1). This protocol, together with an invitation letter, will be provided to the authors of all potentially eligible studies to determine their interest in participating. Data will be accepted in any form, although a standardized format will be sought. If authors are unable to share full individual participant datasets that adhere to our guidelines, efforts will be made to have analyses conducted at the collaborating site, with summary metrics shared to enable a secondary set of analyses based upon summary statistics. Two datasets will be generated for this project: one comprising the urine data collected at a single time-point; the second comprising urine data collected at multiple time-points (which will be used for the change analyses).

### Statistical Analyses

Participants with suspected incomplete 24-hour urine samples (ie, <80% urinary para-aminobenzoic acid recovery [if available], or urinary creatinine <4.0 mmol/day for women or < 6.0 mmol/day for men, or a 24-hour urine collection of <500ml for either sex) and suspected over-collections (ie, urinary creatinine or a urine collection volume >3 standard deviations above the population mean) will be excluded from primary analyses. For all analyses, statistical significance will be set at *P*<0.05. Data will be analyzed using Stata V13.0 (Stata Corp, College Station, TX, USA).

#### Analysis One: Comparison of Existing Equations for Estimating 24-Hour Salt Intake from Spot Urine Samples with 24-Hour Urine Samples, and Assessment of the Degree of Bias According to Key Demographic and Clinical Characteristics

For each individual, the 24-hour sodium excretion (24-hour_sodium_) value (mmol/day) based on the 24-hour urine collection will be calculated as the concentration of sodium in the urine (mmol/L) multiplied by the urinary volume (L/day). The conversion from sodium (mmol/day) to sodium (mg/day) will be made by multiplying by 23, and the conversion from sodium (g/day) to salt (g/day) will be made by multiplying the sodium value by 2.542. Estimated 24-hour salt excretion (24-hour_salt_) from spot urine samples (24-hour_spot_) will be calculated from currently-used estimation equations (ie, Kawasaki [36], Tanaka [33], Mage [30,31], Toft [34], and INTERSALT with and without potassium [32]; see Multimedia Appendix 2). Within-person bias (24-hour_bias­_) will be calculated as 24-hour_spot_- 24-hour_salt_. Pooled summary estimates across studies (24-hour_salt_, 24-hour_spot_, and 24-hour_bias_) will be calculated using inverse-variance weighted fixed effects meta-analyses [44]. In cases where substantial variability across contributing studies is identified based on the I^2^statistic, sensitivity analyses will be conducted using the random effects model according to DerSimonian and Laird [45].

The effects of ethnicity, sex, age, BMI, antihypertensive drug use, health status (diabetes, kidney disease, cardiovascular disease, or cerebrovascular disease), timing of spot urine collection (overnight, morning, afternoon, evening, or timed sample), and 24-hour salt (as estimated by 24-hour urine collection) on the magnitude of bias for each estimation equation will be examined by conducting multiple linear regression. The beta-coefficient resulting from the multiple linear regression will be the difference in mean 24-hour_bias_ for each unit difference for each pre-specified demographic and clinical characteristic. Beta-coefficients and their standard errors for each study will be pooled, and summary effects will be calculated using inverse-variance weighted fixed effects meta-analysis.

Within-trial proportional bias will be assessed by determining the association between level of intake (mean of 24-hour_salt_ and 24-hour_spot_) and 24-hour_bias­_. The regression coefficients will then be meta-analyzed across the cohorts using inverse-variance fixed effects meta-analysis. In cases of substantial variability, random effects models will be used.

#### Analysis Two: Measuring Population Change in Salt Intake Using Spot Urine Samples

Data collected from the same person at two or more time-points at least 1 month apart (paired data), and data from the same population (but different individuals within the population) at two or more time-points (unpaired), will be sought. The capacity of spot urine samples to track population changes in salt intake will be determined by assessing the paired and unpaired data separately in the first instance. At the individual study level, repeated measures analyses of variance will be used for analyses of the paired data, and independent samples t-tests for the unpaired data. Pooled summary estimates across studies will be calculated using inverse-variance weighted fixed effects meta-analyses for paired and unpaired data separately. To maximize the sample size, the unpaired and paired data will also be analyzed together using inverse-variance weighted fixed effects meta-analyses. The effects of covariates (ethnicity, sex, age, BMI, antihypertensive drug use, health status, and timing of spot urine collection) on equation performance will be examined by including each of these covariates in the model. Both unadjusted and adjusted analyses will be undertaken, following prior exploration of the associations between each covariate of interest.

#### Analysis Three: Development of an Equation (Or Equations) that Performs Better than Existing Equations to Estimate Mean Population 24-Hour Urinary Salt Excretion

Sex-specific regression equations estimating 24-hour sodium excretion will be obtained from spot urine sodium excretion, potassium, creatinine, age, BMI, and ethnicity. Inclusion of the specific variables in the models will be assessed using stepwise selection. The final model fit will be assessed using the R^2^value from 5000 samples drawn using bootstrapping. The observed mean 24-hour sodium excretion will be compared with the estimated sodium excretion from the new equation (or equations) at a population level using Pearson’s correlation. Bland Altman plots will also be produced to calculate the mean bias between measured 24-hour sodium excretion and estimated excretion from the spot urine samples using the new equation (or equations) [46].

### Risk of Bias Assessment

Study-level risk of bias will be assessed using the Newcastle-Ottawa Scale [47]. In addition, checks of the individual participant data will be conducted to ensure that the data reflects the methods reported in the original publication [48].

### Leadership and Data Curation

A Steering Committee will be established to lead the initiative, with operational support provided by a Secretariat. The Steering Committee will have final responsibility for scientific outputs and will include a representative from each study that is able to contribute individual participant data, plus members of the Secretariat. Steering Committee members will be responsible for finalizing the protocol and agreeing upon all outputs from the initiative, and will be supported by a statistician that will provide data curation and analysis services. All data provided for the initiative will be held in confidence and used only for the purposes described in this protocol or its subsequent amendments, as agreed upon by the Steering Committee. Steering Committee members will be free to withdraw their data from the initiative at any time, should they choose.

## Results

The most recent search was completed in March 2016 and the results of the search are included as a Multimedia Appendix 3. Briefly, the search identified 538 records with one unpublished (but eligible) study. A total of 430 records were screened, and based on the abstracts 262 did not meet the inclusion criteria. A sample of 100 full-text articles were assessed for eligibility. Following this review, 73 studies were confirmed to be eligible (Multimedia Appendix 4), and a further 68 abstracts were identified that may be eligible for inclusion in the project (Multimedia Appendix 5). Authors from the 73 eligible studies and the 68 abstracts will be invited to contribute data.

## Discussion

Multiple reports now suggest that mean population salt intake can be estimated from spot urine samples, although the robustness of the estimates obtained and the best methods for obtaining them remains unclear for many parts of the world. The recent adoption of spot urine samples for the estimation of mean population salt intake by the WHO, as part of the WHO Stepwise Approach to Risk Factor Surveillance, has placed additional priority on efforts to resolve these and related questions.

The primary objectives of this individual-participant data meta-analysis have been defined on the basis of the most pressing need, and relate to the capacity of equations based upon spot urine samples to provide results that approximate those obtained with 24-hour urine samples. The aims of this study also focus specifically on the capacity of the estimates (based on spot urine samples) to track changes in mean population salt intake over time. Studies published to date that have assessed the validity of using spot urine samples to measure average salt intake have been cross-sectional (ie, urine samples are collected at one time-point) [38-41,49-51]. Therefore, such studies only provide limited inference about the implications of using spot urine samples to monitor change over time.

A number of characteristics regarding populations and individuals might influence the capacity of different equations to provide estimates of mean population salt intake, and these issues are the focus of some of the analyses proposed. Diurnal variation in salt excretion may favor estimates based upon spot urine samples collected at a particular time of day [52]. Likewise, equations may perform differently in hypertensive compared to non-hypertensive patients, both because of reported changes in the diurnal excretion of salt and because drug therapies (eg, diuretics) affect urinary salt excretion [37,49,53-55].

The proposed analyses will also make it possible to quantify or control for the effects of methodological differences in studies, and how these effects impact on the conclusions drawn. For example, studies that use a spot urine sample collected as part of the 24-hour collection may over-estimate the coherence of population estimates based upon spot and 24-hour samples compared to studies in which the spot urine sample was collected on another day entirely. This project will also enable standardization of the 24-hour comparator group (against which estimates based upon spot samples are compared) by applying the same set of quality criteria to the 24-hour urine samples across studies. Even with the application of rigorous quality criteria to the 24-hour samples, the 24-hour standard falls far short of being a true gold standard; even if urine collections are ascertained to be complete, salt excretion is known to vary substantially from day-to-day, and a significant proportion of salt is excreted through nonurinary routes [56]. It is, however, the current standard upon which clinical and public health decisions are made, and represents the only plausible comparator for the proposed analyses.

It is of note that several prior studies have drawn conclusions about the likely value of spot urine samples based upon metrics (such as correlation coefficients) that compare the values obtained for individuals. While high values for such metrics will typically be associated with good correlation at the population level, these are not necessarily the best means for evaluation in this setting since good correlation of data at the individual level is not a prerequisite for obtaining robust population estimates [46].

In summary, many individual studies now exist that have identified equations based upon spot urine samples as a plausible alternative to 24-hour urine samples for the estimation of mean population salt intake. There remain, however, important questions about the best approach to estimate 24-hour excretion from spot urine samples, and the capacity of estimated 24-hour salt excretion derived from spot urine samples to detect changes in population salt intake over time. This systematic review and meta-analysis should resolve much of this uncertainty and provide new data that will allow for specific recommendations to be made about the best way to measure population salt intake using spot urine samples. These findings will be a significant contribution to scientific fields and to public health.

## Appendix

Multimedia Appendix 1 Search terms.

Multimedia Appendix 2 Predictive equations used to estimate 24-hour salt intake (g) from spot urine samples.

Multimedia Appendix 3 PRISMA Diagram.

Multimedia Appendix 4 Identified studies with full text report available.

Multimedia Appendix 5 Identified studies for which full text is unavailable and eligibility is uncertain.

## Abbreviations

BMI: body mass index
NHMRC: National Health and Medical Research Council
WHO: World Health Organization
