# Supplementary material for: Reliable Quantification of the Potential for Equations Based on Spot Urine Samples to Estimate Population Salt Intake: Protocol for a Systematic Review and Meta-Analysis
Source: JMIR Res Protoc. 2016 Sep 21;5(3):e190. doi: 10.2196/resprot.6282 (PMC5052460; doi:10.2196/resprot.6282)
Supplement: Multimedia Appendix 1 [file resprot_v5i3e190_app1.pdf]

MULTIMEDIA APPENDIX 1

Search terms

Medline via Ovid

1. ((24-hr* or 24hr* or 24 hour* or 24hour*) adj5 urin*).tw.
2. ((spot or spot- or casual or random or timed or timed- or nocturnal or overnight or morning or fractional or afternoon or evening or sample) adj5 urin*).tw.
3. Sodium/
4. ((dietar* or chloride* or intake* or excretion*) adj5 salt).tw.
5. ((dietar* or chloride* or intake* or excretion*) adj5 sodium).tw.
6. ((dietar* or chloride* or intake* or excretion*) adj5 Na).tw.
7. 1 and 2
8. 3 or 4 or 5 or 6
9. 7 and 8

PreMedline via Ovid

10. ((24-hr* or 24hr* or 24 hour* or 24hour*) adj5 urin*).tw.
11. ((spot or spot- or casual or random or timed or timed- or nocturnal or overnight or morning or fractional or afternoon or evening or sample) adj5 urin*).tw.
12. Sodium/
13. ((dietar* or chloride* or intake* or excretion*) adj5 salt).tw.
14. ((dietar* or chloride* or intake* or excretion*) adj5 sodium).tw.
15. ((dietar* or chloride* or intake* or excretion*) adj5 Na).tw.
16. 1 and 2
17. 3 or 4 or 5 or 6
18. 7 and 8

Global Health via Ovid

1. ((24-hr* or 24hr* or 24 hour* or 24hour*) adj5 urin*).tw.
2. ((spot or spot- or casual or random or timed or timed- or nocturnal or overnight or morning or fractional or afternoon or evening or sample) adj5 urin*).tw.
3. Sodium/
4. ((dietar* or chloride* or intake* or excretion*) adj5 salt).tw.
5. ((dietar* or chloride* or intake* or excretion*) adj5 sodium).tw.
6. ((dietar* or chloride* or intake* or excretion*) adj5 Na).tw.
7. 1 and 2
8. 3 or 4 or 5 or 6
9. 7 and 8

Embase

1. (24hr* NEAR/5 urin*):ab,ti AND [embase]/lim
2. 24:ab,ti AND (hour* NEAR/5 urin*):ab,ti AND [embase]/lim
3. 24:ab,ti AND (hr* NEAR/5 urin*):ab,ti AND [embase]/lim
4. (24hour* NEAR/5 urin*):ab,ti AND [embase]/lim
5. (spot NEAR/5 urin*):ab,ti AND [embase]/lim

6. (spot- NEAR/5 urin*):ab,ti AND [embase]/lim
7. (casual NEAR/5 urin*):ab,ti AND [embase]/lim
8. (random NEAR/5 urin*):ab,ti AND [embase]/lim
9. (timed NEAR/5 urin*):ab,ti AND [embase]/lim
10. (timed- NEAR/5 urin*):ab,ti AND [embase]/lim
11. (overnight NEAR/5 urin*):ab,ti AND [embase]/lim
12. (nocturnal NEAR/5 urin*):ab,ti AND [embase]/lim
13. (morning NEAR/5 urin*):ab,ti AND [embase]/lim
14. (afternoon NEAR/5 urin*):ab,ti AND [embase]/lim
15. (fractional NEAR/5 urin*):ab,ti AND [embase]/lim
16. (evening NEAR/5 urin*):ab,ti AND [embase]/lim
17. (sample NEAR/5 urin*):ab,ti AND [embase]/lim
18. 'sodium'/exp AND [embase]/lim
19. (dietar* NEAR/5 salt):ab,ti AND [embase]/lim
20. (chloride* NEAR/5 salt):ab,ti AND [embase]/lim
21. (intake* NEAR/5 salt):ab,ti AND [embase]/lim
22. (excretion* NEAR/5 salt):ab,ti AND [embase]/lim
23. (dietar* NEAR/5 sodium):ab,ti AND [embase]/lim
24. (chloride* NEAR/5 sodium):ab,ti AND [embase]/lim
25. (intake* NEAR/5 sodium):ab,ti AND [embase]/lim
26. (excretion* NEAR/5 sodium):ab,ti AND [embase]/lim
27. (dietar* NEAR/5 na):ab,ti AND [embase]/lim
28. (chloride* NEAR/5 na):ab,ti AND [embase]/lim
29. (intake* NEAR/5 na):ab,ti AND [embase]/lim
30. (excretion* NEAR/5 na):ab,ti AND [embase]/lim
31. #1 OR #2 OR #3 OR #4
32. #5 OR #6 OR #7 OR #8 OR #9 OR #10 OR #11 OR #12 OR #13 OR #14 OR #15 OR #16 OR #17
33. #31 AND #32
34. #18 OR #19 OR #20 OR #21 OR #22 OR #23 OR #24 OR #25 OR #26 OR #27 OR #28 OR #29 OR #30
35. #33 AND #34

Cochrane

1. ((24-hr* or 24hr* or 24 hour* or 24hour*) adj5 urin*).tw.
2. ((spot or spot- or casual or random or timed or timed- or nocturnal or overnight or morning or fractional or afternoon or evening or sample) adj5 urin*).tw.
3. Sodium/
4. ((dietar* or chloride* or intake* or excretion*) adj5 salt).tw.
5. ((dietar* or chloride* or intake* or excretion*) adj5 sodium).tw.
6. ((dietar* or chloride* or intake* or excretion*) adj5 Na).tw.
7. #1 and #2
8. #3 or #4 or #5 or #6
9. #7 and #8
