# Supplementary material for: Reliable Quantification of the Potential for Equations Based on Spot Urine Samples to Estimate Population Salt Intake: Protocol for a Systematic Review and Meta-Analysis
Source: JMIR Res Protoc. 2016 Sep 21;5(3):e190. doi: 10.2196/resprot.6282 (PMC5052460; doi:10.2196/resprot.6282)
Supplement: Multimedia Appendix 2 [file resprot_v5i3e190_app2.pdf]

MULTIMEDIA APPENDIX 2

Predictive equations used to estimate 24 hour salt intake (g) from spot urine samples

**Kawasaki [1]**	
Male	2.54 ÷ 1000 × 23× 16.3 × {spot Na (mmol/l)/[spot Cr (mg/dl) x 10] × [− 12.63 × age (years) + 15.12 × weight (kg) +7.39 × height (cm) − 79.9]}$^{0.5}$
Female	2.54 ÷ 1000 × 23× 16.3 × {spot Na (mmol/l)/[spot Cr (mg/dl) x 10] × [− 4.72 × age (years) + 8.58 × weight (kg) + 5.09 × height (cm) − 74.5]}$^{0.5}$
**Tanaka [2]**	2.54÷ 1000 × 23× 21.98 × {spot Na (mmol/l)/[spot Cr (mg/dl) × 10] ×[− 2.04 × age (years) + 14.89×weight (kg) + 16.14 × height (cm) − 2244.45]}$^{0.392}$
**Mage [3, 4]**	
Male	2.54 ÷ 1000 × 23 × [spot Na (mmol/L)/(spot Cr (mg/dL) × 10)] ×{0.00179 × [140 − age (y)] × [weight (kg)$^{1.5}$ × height (cm)$^{0.5}$] × [[1 + 0.18 × A] × [1.366 − 0.0159 × BMI (kg/m^2)]]}, where A is African American or black race = 1, other race = 0.
Female	2.54 ÷ 1000 × 23 × [spot Na (mmol/L)/(spot Cr (mg/dL) × 10)] × {0.00163 × [140 − age (y)] × [weight (kg)$^{1.5}$ × height (cm)$^{0.5}$] × [[1 + 0.18 × A] × [1.429 − 0.0198 × BMI (kg/m^2)]]}, where A is African American or black race = 1, other race = 0.
**Toft [5]**	
Male	2.54 ÷ 1000 × 23 × 33.56 × {spot Na (mmol/L)/spot Cr (mg/dL) × [− 7.54 × age (years) + 14.15 × weight (kg) + 3.48 height (cm) + 423.15]}$^{0.345}$
Female	2.54 ÷ 1000 × 23 × 52.65 × {spot Na (mmol/L)/spot Cr (mg/dL) × [−6.13 × age (years) + 9.97 × weight (kg) + 2.45 × height (cm) +342.73]}$^{0.196}$
**INTERSALT with Potassium [6]**	
**North America**	
**Male**	2.54 ÷ 1000 × 23 × {25.46 + [0.46 × spot Na (mmol/L)] − [2.75 × spot Cr (mmol/L)] − [0.13 × spot K (mmol/L)] + [4.10 × BMI (kg/m^2)] + [0.26 × age (y)]}
**Female**	2.54 ÷ 1000 × 23 × {5.07 + [0.34 × spot Na (mmol/L)] − [2.16 × spot Cr (mmol/L)] − [0.09 × spot K (mmol/L)] + [2.39 × BMI (kg/m^2)] + [2.35 × age (y)] − [0.03 × age^2 (y)]}
**Northern Europe**	
Male	2.54 ÷ 1000 × 23× {23.17 + [0.46 × spot Na (mmol/L)] − [2.75 × spot Cr (mmol/L)] − [0.13 × spot K (mmol/L)] + [4.10 × BMI (kg/m^2)] + [0.26 × age (y)]}
Female	2.54 ÷ 1000 × 23 × {15.73 + [0.34 × spot Na (mmol/L)] − [2.16 × spot Cr (mmol/L)] − [0.09 × spot K (mmol/L)] + [2.39 × BMI (kg/m^2)] +

	[2.35 × age (y)] – [0.03 × age^2 (y)]}
**Eastern Europe**	
Male	2.54 ÷ 1000 × 23 × {39.56 + [0.46 × spot Na (mmol/L)] − [2.75 × spot Cr (mmol/L)] − [0.13 × spot K (mmol/L)] + [4.10 × BMI (kg/m^2)] + [0.26 × age (y)]}
Female	2.54 ÷ 1000 × 23× {16.94 + [0.34 × spot Na (mmol/L)] − [2.16 × spot Cr (mmol/L)] − [0.09 × spot K (mmol/L)] + [2.39 × BMI (kg/m^2)] + [2.35 × age (y)] – [0.03 × age2 (y)]}
**Southern Europe**	
Male	2.54 ÷ 1000 × 23× {23.08 + [0.46 × spot Na (mmol/L)] − [2.75 × spot Cr (mmol/L)] − [0.13 × spot K (mmol/L)] + [4.10 × BMI (kg/m^2)] + [0.26 × age (y)]}
Female	2.54 ÷ 1000 × 23× {23.79 + [0.34 × spot Na (mmol/L)] − [2.16 × spot Cr (mmol/L)] − [0.09 × spot K (mmol/L)] + [2.39 × BMI (kg/m^2)] + [2.35 × age (y)] – [0.03 × age^2 (y)]}
**Western Europe**	
Male	2.54 ÷ 1000 × 23 × {17.05 + [0.46 × spot Na (mmol/L)] − [2.75 × spot Cr (mmol/L)] − [0.13 × spot K (mmol/L)] + [4.10 × BMI (kg/m^2)] + [0.26 × age (y)]}
Female	2.54 ÷ 1000 × 23 × {12.82 + [0.34 × spot Na (mmol/L)] − [2.16 × spot Cr (mmol/L)] − [0.09 × spot K (mmol/L)] + [2.39 × BMI (kg/m^2)] + [2.35 × age (y)] – [0.03 × age^2 (y)]}
**INTERSALT without Potassium [6]**	
**North America**	
Male	2.54 ÷ 1000 × 23 × {23.51 + [0.45 × spot Na (mmol/L)] − [3.09 × spot Cr (mmol/L)] + [4.16 × BMI (kg/m^2)] + [0.22 × Age (y)]}
Female	2.54 ÷ 1000 × 23 × {3.74 + [0.33 × spot Na (mmol/L)] − [2.44 × spot Cr (mmol/L)] + [2.42 × BMI (kg/m^2)] + [2.34 × Age(y) − [0.03× Age^2(y)]}
**Northern Europe**	
Male	2.54 ÷ 1000 × 23 × {20.93 + [0.45 × spot Na (mmol/L)] − [3.09 × spot Cr (mmol/L)] + [4.16 × BMI (kg/m^2)] + [0.22 × Age (y)]}
Female	2.54 ÷ 1000 × 23 × {14.47 + [0.33 × spot Na (mmol/L)] − [2.44 × spot Cr (mmol/L)] + [2.42 × BMI (kg/m^2)] + [2.34 × Age(y) − [0.03× Age^2(y)]}
**Eastern Europe**	
Male	2.54 ÷ 1000 × 23 × {39.58 + [0.45 × spot Na (mmol/L)] − [3.09 × spot Cr (mmol/L)] + [4.16 × BMI (kg/m^2)] + [0.22 × Age (y)]}
Female	2.54 × 23 ÷ 1000 × {17.02 + [0.33 × spot Na (mmol/L)] − [2.44 × spot Cr (mmol/L)] + [2.42 × BMI (kg/m^2)] + [2.34 × Age(y) − [0.03× Age^2(y)]}
**Southern Europe**	
Male	2.54 ÷ 1000 × 23 × {20.86 + [0.45 × spot Na (mmol/L)] − [3.09 × spot Cr (mmol/L)] + [4.16 × BMI (kg/m^2)] + [0.22 × Age (y)]}
Female	2.54 ÷ 1000 × 23× {21.98 + [0.33 × spot Na (mmol/L)] − [2.44 × spot Cr (mmol/L)] + [2.42 × BMI (kg/m^2)] + [2.34 × Age(y) − [0.03× Age^2(y)]}

**Western Europe**	
Male	2.54 ÷ 1000 × 23 × {14.6 + [0.45 × spot Na (mmol/L)] − [3.09 × spot Cr (mmol/L)] + [4.16 × BMI (kg/m^2)] + [0.22 × Age (y)]}
Female	2.54 ÷ 1000 × 23 × {11.38 + [0.33 × spot Na (mmol/L)] − [2.44 × spot Cr (mmol/L)] + [2.42 × BMI (kg/m^2)] + [2.34 × Age(y) − [0.03× Age^2(y)]}

REFERENCES

1. Kawasaki T, Itoh K, Uezono K, Sasaki H. A simple method for estimating 24 h urinary sodium and potassium excretion from second morning voiding urine specimen in adults. Clin Exp Pharmacol Physiol 1993; 20: 7-14. PMID 8432042
2. Tanaka T, Okamura T, Miura K, Kadowaki T, Ueshima H, Nakagawa H, Hashimoto T. A simple method to estimate populational 24-h urinary sodium and potassium excretion using a casual urine specimen. J Hum Hypertens 2002; 16: 97-103. PMID 11850766 DOI 10.1038/sj.jhh.1001307
3. Mage DT, Allen RH, Kodali A. Creatinine corrections for estimating children's and adult's pesticide intake doses in equilibrium with urinary pesticide and creatinine concentrations. J Expo Sci Environ Epidemiol 2008; 18: 360-8. PMID 17878925 DOI 10.1038/sj.jes.7500614
4. Huber DR, Blount BC, Mage DT, Letkiewicz FJ, Kumar A, Allen RH. Estimating perchlorate exposure from food and tap water based on US biomonitoring and occurrence data. J Expo Sci Environ Epidemiol 2011; 21: 395-407. PMID 20571527 DOI 10.1038/jes.2010.31
5. Toft U, Cerqueira C, Andreasen AH, Thuesen BH, Laurberg P, Ovesen L, Perrild H, Jorgensen T. Estimating salt intake in a caucasian population: Can spot urine substitute 24-hour urine samples? Eur J Prev Cardiol 2014; 21: 1300-1307. PMID 23559538
6. Brown IJ, Dyer AR, Chan Q, Cogswell ME, Ueshima H, Stamler J, Elliott P. Estimating 24-hour urinary sodium excretion from casual urinary sodium concentrations in western populations: the INTERSALT Study. Am J Epidemiol 2013; 177: 1180-1192. PMID 23673246
