# Supplementary figures and images for: Reliable Quantification of the Potential for Equations Based on Spot Urine Samples to Estimate Population Salt Intake: Protocol for a Systematic Review and Meta-Analysis
Source: JMIR Res Protoc. 2016 Sep 21;5(3):e190. doi: 10.2196/resprot.6282 (PMC5052460; doi:10.2196/resprot.6282)

## Multimedia Appendix 3

Figure: PRISMA Diagram

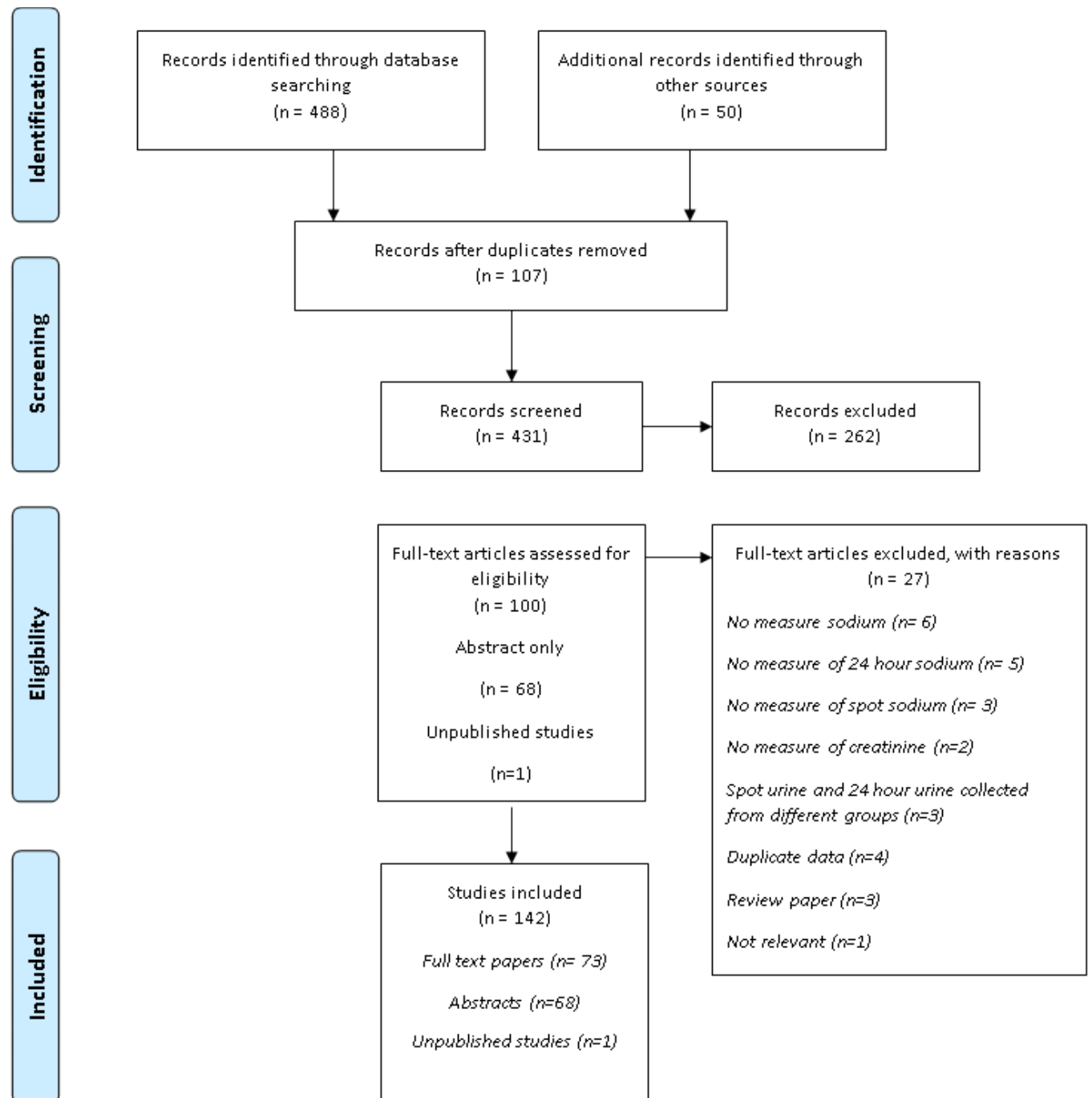

Supplement: Multimedia Appendix 3 [file resprot_v5i3e190_app3.pdf]
