# Supplementary material for: Reliable Quantification of the Potential for Equations Based on Spot Urine Samples to Estimate Population Salt Intake: Protocol for a Systematic Review and Meta-Analysis
Source: JMIR Res Protoc. 2016 Sep 21;5(3):e190. doi: 10.2196/resprot.6282 (PMC5052460; doi:10.2196/resprot.6282)
Supplement: Multimedia Appendix 4 [file resprot_v5i3e190_app4.pdf]

Multimedia Appendix 4

Table: Identified studies with full text report available

First author Year	Country	Sample size	Study population	Age[#]	Spot urine timing				Timing of spot urine and 24 hour collection	
					Random/ not reported	Morning	Afternoon	Evening/ overnight	Dependent	Independent
Bankir L 2008 [1]	Seychelles Islands	325	Eastern African descent	46.4±11.5		X		X	X	
Brown IJ 2013 [2]	European and North American	5693	Random sample	20-59	X					X
Chen WY 1975 [3]	China	188	Normotensive and hypertensive	24-72	X					X
Cholongitas E 2013 [4]	Greece	126	Patients with decompensated cirrhosis and ascites without intrinsic renal disease	56±12	X					X
Cogswell ME 2013 [5]	United States	407	Healthy	18-39		X	X	X	X	
Ding J 1983 [6]	China	20	Healthy hospital staff	30-50	X				X	
Doenyas-Barak K 2015 [7]	Israel	48	Healthy volunteers	46.6±14.0		X	X	X	X	
Dyer A 1987 [8]	United States	107	Treated hypertensives	41-80		X	X	X	X	
Dyer A 1998 [9]	United States	120	General population	27-64		X	X	X	X	
El-Bokl MA	Egypt	40	Patients with liver	50±5	X				-	

2009 [10]			cirrhosis and ascites							
Enkhtungalag B [unpublished]	Mongolia	1027	General population	25-64		X		X	-	
Ford EG 1987 [11]	United States	6	Children admitted to the pediatric surgical service for traumatic disease	1-13	X				X	
Green TJ 1994 [12]	Canada	8	Male volunteers	25.4±4		X				X
Haga M 2010 [13]	Japan	79	Infants	2-5				X	X	
Han W 2015 [14]	China	222	Hypertensive patients	58.4±14.5		X	X		X	
He J 1993 [15]	China	63	Liangshan Yi farmers (rural) and urban residents (staff of Yi Migrant Study)	19-55				X	X	
He J 2009 [16]	China	1906	Pre-hypertensive or stage 1 hypertension	39.3±9.6				X		-
He J 2012 [17]	China	487	Pre-hypertensive or stage 1 hypertension	44.3±8.7				X	Not reported	
Hollister AS 1986 [18]	United States	14	Normotensive	33±2	X					X
Hong YH 2010 [19]	Malaysia	30	Urinary stone-formers	-		X				X
Hunsballe JM 1998 [20]	Denmark	33	Patients with primary monosymptomatic nocturnal enuresis and Healthy subjects	15-37	X			X	X	

Ilich JZ<br>2009 [21]	United States	143	Healthy women	30-79		X				X
Imai E<br>2011 [22]	Japan	136	Patients with chronic kidney disease	Male: 67.5±12<br>Female: 69.1±10.3		X			X	
Iwahori T<br>2014 [23]	Japan	48	General population	39.9	X	X			X	
Jeffery PB<br>2013 [24]	Australia	948	General population	56.5±13.6		X		X	X	
Ji C<br>2013 [25]	Britain (European White, West African Black, South Asian), Italy	1063	General population and factory workers	32-75		X				X
Kamata K<br>2002 [26]	Japan	149	Healthy subjects	Men: 35±14.4; women: 49±15.8		X			X	
Kang S<br>2012 [27]	Korea	305	Patients with chronic kidney disease	55.7±14.1		X	X	X		X
Kara PS<br>2013 [28]	Turkey	42	Normotensive and hypertensive	hypertensive: 55.8±8.4; normotensives: 49.3±5.4		X	X	X	X	
Kawamura M<br>2010 [29]	Japan	100	Hypertensive	Men: 58 ± 14; Women: 61 ±12		X				X
Kawamura M<br>2012 [30]	Japan	24	Hypertensive	59±16	X	X				X
Kawasaki T<br>1982 [31]	Japan	242	Healthy	20-63				-		
Kirkland JL<br>1983 [32]	England	45	General population	25-80	X				X	

Kirkpantur A 2013 [33]	Turkey	114	Newly diagnosed type 2 diabetes	45.2±10.2		-	
Knuiman JT 1988 [34]	Netherlands	28	School children	8-9		X	X
Koo H 2015 [35]	Korea	204	General population	-	X		X
Liu K 1979 [36]	United States	73	6th-8th grade school children	-		X	X
Liu K 1979 [37]	United States	142	Male business and administrative personnel	-	X	X	X
Liu K 1984 [38]	United States	189	Men with diastolic blood pressure 80-90 mmHg and school children in the 6th to 8th grades	38.2±4.4; 12.2±0.9		-	
Liu LS 1986 [39]	China	49	Healthy male doctors or technicians	30-50		X	X
Liu LS 1987 [40]	China	50	Normotensive male employees of Fu Wai hospital in Beijing, China	27-50	X		X
Luft F 1982 [41]	United States	24	Male students or employees at Indiana University	19-32		X	X
Luft F 1982 [42]	United States	43	Students and employees at Indiana University	19-54		X	X
Luft F 1983 [43]	United States	24	Students and employees at Indiana University	22-62	X	X	X

Luft FC 1984 [44]	United States	153	Normotensive school-age identical twin pairs and their families	Fathers: 40±2.5 Mothers: 37±1.9 Children: 11±0.2	X			X	X	
Mann S 2010 [45]	United States	81	General population	59.2±12.2	X	X	X		X	X
McLean R 2014 [46]	New Zealand	98	Healthy	18-65	X				X	
Mente A 2014 [47]	India, China, Colombia, Argentina, Brazil, Malaysia, South Africa, Turkey, Canada, Sweden, United Arab Emirates	1083	General population	35-70		X			X	
Micheli E T 2003 [48]	Brazil	31	General population	6-17				X	X	
Mill JG 2012 [49]	Brazil	109	Healthy	30-74	X			X	X	
Milne FJ 1980 [50]	South Africa	97	Normotensive and hypertensive	-		X	X	X	X	
Mizehoun-Adissoda C 2015 [51]	West Africa	354	Healthy	25-64		X			X	
Ogawa M 1986 [52]	Japan	16	Normotensive and pre-hypertensive	19-65		X			X	

Pan WH 1994 [53]	Taiwan	39	Healthy	24			X	X	
Parsons CL 2005 [54]	United States	48	New untreated female patients with interstitial cystitis (IC) and control patients without IC symptoms	-	X				X
Peco-Antić A 2009 [55]	Serbia	56	Patients with chronic glomerulopathy	4-19	X		X	X	
Pietinen PI 1976 [56]	United States	19	Young healthy normotensive individuals	21-38	X		X	X	
Pietinen PI 1979 [57]	United States	50	Healthy volunteers	20-40			X	X	
Poulter N 1984 [58]	Kenya	32	Volunteers	-	X				X
Rhee M 2014 [59]	Korea	325	General population	Group 1: 52.4 ± 11.1<br>Group 2: 51.0 ± 10.9	X	X	X	X	
Tanaka T 2002 [60]	Japan	927	General population	20-59	X				X
Tochikubo O 1986 [61]	Japan	103	Hypertenvie and normotensive high school students	15-18			X	X	
Toft U 2013 [62]	Denmark	473	General population	28-74	X				X
Trotter A 1996 [63]	Germany	70	Newborn infants	Postmenstrual median age 25-42 weeks	X			X	
Vanacor R	Brazil	60	Healthy	33.7±13.5	X			X	

2008 [64]									
Watson RL 1970 [65]	United States	100	General population	-	X			X	
Webster J 2016 [66]	Samoa	293	General population	36.4±15.4		X		X	
Whiting SJ 1998 [67]	Canada	10	Healthy women	20-30		X			X
Wolf JP 1984 [68]	France	151	Healthy volunteers	20-68		X			-
Yamasue K 2006 [69]	Japan	250	Healthy adults	52.7±15.9			X	X	
Yamauchi T 1994[70]	Japan	22	School children	6-11			X	X	
Yamori Y 1982 [71]	Japan	16	Healthy medical student volunteers	22-24		X	X	X	
Ye Z 1986	China	48	University students	-			X	X	

#presented as range or mean±SD

REFERENCES

1. Bankir L, Bochud M, Maillard M, Bovet P, Gabriel A, Burnier M. Nighttime Blood Pressure and Nocturnal Dipping Are Associated With Daytime Urinary Sodium Excretion in African Subjects. Hypertension 2008; 51: 891-898. PMID 18316653 DOI 10.1161/hypertensionaha.107.105510
2. Brown IJ, Dyer AR, Chan Q, Cogswell ME, Ueshima H, Stamler J, Elliott P. Estimating 24-hour urinary sodium excretion from casual urinary sodium concentrations in western populations: the INTERSALT Study. Am J Epidemiol 2013; 177: 1180-1192. PMID 23673246
3. Chen WY, Hsieh BS, Cheng JT. Plasma renin activity and sodium excretion in normal and hypertensive Chinese. Taiwan Yi Xue Hui Za Zhi 1975; 74: 525-533. PMID 1063251
4. Cholongitas E, Goulis J, Arsos G, Birtsou C, Nakouti T, Papadopoulou S, Chalevas P, Karakatsanis K, Akriviadis E. Association Between Ratio of Sodium to Potassium in Random Urine Samples and Renal Dysfunction and Mortality in Patients With Decompensated Cirrhosis. Clin Gastroenterol Hepatol 2013; 11: 862-867. PMID 23403009 DOI 10.1016/j.cgh.2013.02.005
5. Cogswell M, Wang CY, Chen TC, Pfeiffer C, Elliott P, Gillespie C, Carriquiry A, Sempos C, Liu K, Perrine C, Swanson C, Caldwell K, Loria C. Validity of predictive equations for 24-hour urine sodium excretion in young black and other adults. Circulation 2013; 127.
6. Ding J, Liu L, D Z. Urinary Sodium and Aldosterone excretion of different time periods in normal subjects and their relatioship to 24 hr total excretion. Acta Academiae Medicinae Sinicae 1983; 3: 193-95.
7. Doenyas-Barak K, Beberashvili I, Bar-Chaim A, Averbukh Z, Vogel O, Efrati S. Daily sodium and potassium excretion can be estimated by scheduled spot urine collections. Nephron 2015; 130: 35-40. PMID 25998865
8. Dyer AR, Stamler R, Grimm R, Stamler J, Berman R, Gosch FC, Emidy LA, Elmer P, Fishman J, Van Heel N, Civinelli G. Do hypertensive patients have a different diurnal pattern of electrolyte excretion? Hypertension 1987; 10: 417-424. PMID 3653970
9. Dyer A, Martin G, Burton W, Levin M, Stamler J. Blood pressure and diurnal variation in sodium, potassium, and water excretion. J Hum Hypertens 1998; 12: 363-371. PMID 9705037
10. El-Bokl MA, Senousy BE, El-Karmouty KZ, Mohammed I, Mohammed SM, Shabana SS, Shalaby H. Spot urinary sodium for assessing dietary sodium restriction in cirrhotic ascites. World J Gastroenterol 2009; 15: 3631-5. PMID 19653340
11. Ford EG, Jennings LM, Andrassy RJ. 'Spot' urine collections do not accurately represent 24-hour sodium excretion in surgically stressed children. Pediatr Emerg Care 1987; 3: 231-234. PMID 3432097
12. Green TJ, Whiting SJ. Potassium bicarbonate reduces high protein-induced hypercalciuria in adult men. Nutrition Research 1994; 14: 991-1002. DOI 10.1016/S0271-5317(05)80253-2
13. Haga M, Sakata T. Daily salt intake of healthy Japanese infants of 3-5 years based on sodium excretion in 24-hour urine. J Nutr Sci Vitaminol (Tokyo) 2010; 56: 305-310. PMID 21228501
14. Han W, Sun N, Chen Y, Wang H, Xi Y, Ma Z. Validation of the spot urine in evaluating

24-hour sodium excretion in Chinese hypertension patients. Am J Hypertens 2015; 28: 1368-1375. PMID 26009166

15. He J, Klag MJ, Whelton PK, Chen JY, Mo JP, Qian MC, Coresh J, Mo PS, He GQ. Agreement between overnight and 24-hour urinary cation excretions in Southern Chinese men. Am J Epidemiol 1993; 137: 1212-1220. PMID 8322762
16. He J, Gu D, Chen J, Jaquish CE, Rao DC, Hixson JE, Chen J, Duan X, Huang J, Chen C, Kelly TN, Bazzano LA, Whelton PK. Gender Difference in Blood Pressure Responses to Dietary Sodium Intervention in the GenSalt Study. J Hypertens 2009; 27: 48-54. PMID 19145767
17. He J, Gu D, Zhao Q, Bazzano LA, Huang J, Li J, Lu F, Mu J, Rice T, Rao DC, Chen CS, Hamm LL, Chen J. Reproducibility of blood pressure responses to dietary sodium and potassium interventions: The GenSalt Study. Circulation 2012; 125.
18. Hollister AS, Tanaka I, Imada T. Sodium loading and posture modulate human atrial natriuretic factor plasma levels. Hypertension 1986; 8: II106-II111. PMID 2941366
19. Hong YH, Dublin N, Razack AH, Mohd MA, Husain R. Twenty-four hour and spot urine metabolic evaluations: correlations versus agreements. Urology 2010; 75: 1294-1298. PMID 19914693
20. Hunsballe J, Hansen T, Rittig S, Pedersen EB, Djurhuus J. The efficacy of DDAVP is related to the circadian rhythm of urine output in patients with persisting nocturnal enuresis. Clin Endocrinol (Oxf) 1998; 49: 793-801. PMID 10209568
21. Ilich JZ, Blanuša M, Orlić ŽC, Orct T, Kostial K. Comparison of calcium, magnesium, sodium, potassium, zinc, and creatinine concentration in 24-h and spot urine samples in women. Clin Chem Lab Med 2009; 47: 216-221. PMID 19191729
22. Imai E, Yasuda Y, Horio M, Shibata K, Kato S, Mizutani Y, Imai J, Hayashi M, Kamiya H, Oiso Y, Murohara T, Maruyama S, Matsuo S. Validation of the equations for estimating daily sodium excretion from spot urine in patients with chronic kidney disease. Clin Exp Nephrol 2011; 15: 861-867. PMID 21904907 DOI 10.1007/s10157-011-0523-0
23. Iwahori T, Ueshima H, Miyagawa N, Ohgami N, Yamashita H, Ohkubo T, Murakami Y, Shiga T, Miura K. Six random specimens of daytime casual urine on different days are sufficient to estimate daily sodium/potassium ratio in comparison to 7-day 24-h urine collections. Hypertens Res 2014; 37: 765-771. PMID 24718298 DOI 10.1038/hr.2014.76
24. Jeffery P, Land MA, Riddell L, Shaw J, Webster J, Chalmers J, Smith W, Flood V, Woodward M, Neal B, Nowson C. Correlation between 24-hour and spot/ void urine samples for the purpose of population salt intake assessment. Ann Nutr Metab 2013; 63: 1477.
25. Ji C, Miller M, Venezia A, Strazzullo P, Cappuccio F. Comparisons of spot vs 24-h urine samples for estimating population salt intake: validation study in two independent samples of adults in Britain and Italy. Nutr Metab Cardiovasc Dis 2014; 24: 140-147. PMID 24119990
26. Kamata K, Tochikubo O. Estimation of 24-h urinary sodium excretion using lean body mass and overnight urine collected by a pipe-sampling method. J Hypertens 2002; 20: 2191-2197. PMID 12409957
27. Kang SS, Kang EH, Kim SO, Lee MS, Hong CD, Kim SB. Use of mean spot urine sodium concentrations to estimate daily sodium intake in patients with chronic kidney disease. Nutrition 2012; 28: 256-261. PMID 21996048

28. Kara PS, Erkoc R, Soyoral YU, Begenik H, Aldemir MN. Correlation of 24-hour urine sodium, potassium and calcium measurements with spot urine. European Journal of General Medicine 2013; 10: 20-25.
29. Kawamura M, Hashimoto T, Owada M, Sugawara T. The influence of posture on the estimation of daily salt intake by the second morning urine method. Hypertens Res 2010; 33: 505-510. PMID 20203686
30. Kawamura M, Ohmoto A, Hashimoto T, Yagami F, Owada M, Sugawara T. Second morning urine method is superior to the casual urine method for estimating daily salt intake in patients with hypertension. Hypertens Res 2012; 35: 611-616. PMID 22297479
31. Kawasaki T, Ueno M, Uezono K. Average urinary excretion of sodium in 24 hours can be estimated from a spot-urine specimen. Jpn Circ J 1982; 46: 948-953. PMID 7109212
32. Kirkland JL, Lye M, Levy DW, Banerjee AK. Patterns of urine flow and electrolyte excretion in healthy elderly people. Br Med J (Clin Res Ed) 1983; 287: 1665-7. PMID 6416541
33. Kirkpantur A, Afsar B. Baseline demographic, clinical and laboratory parameters related with 24 hour urinary sodium excretion in newly diagnosed patients with type 2 diabetes. Nephrology Dialysis Transplantation 2012; 27: ii169.
34. Knuiman JT, van Poppel G, Burema J, van der Heijden L, Hautvast JG. Multiple overnight urine collections may be used for estimating the excretion of electrolytes and creatinine. Clin Chem 1988; 34: 135-8. PMID 3338143
35. Koo H, Lee S, Kim J. Evaluation of random urine sodium and potassium compensated by creatinine as possible alternative markers for 24 hours urinary sodium and potassium excretion. Ann Lab Med 2015; 35: 238-241. PMID 25729727
36. Liu K, Cooper R, Soltero I, Stamler J. Variability in 24-hour urine sodium excretion in children. Hypertension 1979; 1: 631-636. PMID 575526
37. Liu K, Dyer AR, Cooper RS, Stamler R, Stamler J. Can overnight urine replace 24-hour urine collection to asses salt intake? Hypertension 1979; 1: 529-536. PMID 541044
38. Liu K, Stamler J. Assessment of sodium intake in epidemiological studies on blood pressure. Ann Clin Res 1984; 16: 49-54. PMID 6336025
39. Liu L, Zheng D, Lai S. Variability in 24-hour urine sodium excretion in Chinese adults. Chin Med J (Engl) 1986; 99: 424-426. PMID 3100177
40. Liu L, Deyu Z, Lue J, Liao Y, Liu K, Stamler J. Variability of urinary sodium and potassium excretion in north Chinese men. J Hypertens 1987; 5: 331-335. PMID 3611781
41. Luft FC, Fineberg NS, Sloan RS. Overnight urine collections to estimate sodium intake. Hypertension 1982; 4: 494-8. PMID 6891373
42. Luft FC, Fineberg NS, Sloan RS. Estimating dietary sodium intake in individuals receiving a randomly fluctuating intake. Hypertension 1982; 4: 805-8. PMID 7141607
43. Luft FC, Sloan RS, Fineberg NS, Free AH. The utility of overnight urine collections in assessing compliance with a low sodium intake diet. JAMA 1983; 249: 1764-1768. PMID 6827767
44. Luft FC, Miller JZ, Fineberg NS, Daugherty SA, Christian JC, Weinberger MH. Estimation of dietary sodium intake in children. Pediatrics 1984; 73: 318-323. PMID 6538331
45. Mann SJ, Gerber LM. Estimation of 24-hour sodium excretion from spot urine

samples. Journal of Clinical Hypertension 2010; 12: 174-180. PMID 20433530
46. McLean R, Williams S, Mann J. Monitoring population sodium intake using spot urine samples: validation in a New Zealand population. J Hum Hypertens 2014; 28: 657-662. PMID 24573130 DOI 10.1038/jhh.2014.10
47. Mente A, O'Donnell MJ, Dagenais G, Wielgosz A, Lear SA, McQueen MJ, Jiang Y, Xingyu W, Jian B, Calik KBT. Validation and comparison of three formulae to estimate sodium and potassium excretion from a single morning fasting urine compared to 24-h measures in 11 countries. J Hypertens 2014; 32: 1005-1015. PMID 24569420
48. Micheli ET, Rosa AA. Estimation of sodium intake by urinary excretion and dietary records in children and adolescents from Porto Alegre, Brazil: a comparison of two methods. Nutrition Research 2003; 23: 1477-1487. DOI 10.1016/S0271-5317(03)00157-X
49. Mill J, da Silva A, Baldo M, Molina M, Rodrigues S. Correlation between sodium and potassium excretion in 24-and 12-h urine samples. Braz J Med Biol Res 2012; 45: 799-805. PMID 22782553
50. Milne FJ, Gear JS, Laidley L, Ritchie M, Schultz E. Spot urinary electrolyte concentrations and 24 hour excretion. Lancet 1980; 2: 1135. PMID 6107745
51. Mizéhoun‐Adissoda C, Houehanou C, Chianéa T, Dalmay F, Bigot A, Preux PM, Bovet P, Houinato D, Desport JC. Estimation of Daily Sodium and Potassium Excretion Using Spot Urine and 24‐Hour Urine Samples in a Black Population (Benin). J Clin Hypertens (Greenwich) 2015. PMID 26530545
52. Ogawa M. Feasibility of overnight urine for assessing dietary intakes of sodium, potassium, protein and sulfur amino acids in field studies. Jpn Circ J 1986; 50: 595-600. PMID 3773224
53. Pan WH, Chen JY, Chen YC, Tsai WY. Diurnal electrolyte excretion pattern affects estimates of electrolyte status based on 24-hour, half-day, and overnight urine. Chin J Physiol 1994; 37: 49-53. PMID 7956513
54. Parsons CL, Greene RA, Chung M, Stanford EJ, Singh G. Abnormal urinary potassium metabolism in patients with interstitial cystitis. J Urol 2005; 173: 1182-1185. PMID 15758737
55. Peco-Antić A, Marinković J, Kruščić D, Paripović D. Circadian rhythms of diuresis, proteinuria and natriuresis in children with chronic glomerular disease. Pediatr Nephrol 2009; 24: 1165-1172. PMID 19184117 DOI 10.1007/s00467-008-1110-9
56. Pietinen PI, Findley TW, Clausen JD. Studies in community nutrition: estimation of sodium output. Prev Med 1976; 5: 400-407. PMID 987585
57. Pietinen PI, Wong O, Altschul AM. Electrolyte output, blood pressure, and family history of hypertension. Am J Clin Nutr 1979; 32: 997-1005. PMID 433826
58. Poulter N, Khaw KT, Hopwood BE, Mugambi M, Peart WS, Rose G, Sever PS. Blood pressure and associated factors in a rural Kenyan community. Hypertension 1984; 6: 810-3. PMID 6335133
59. Rhee M, Kim J, Shin S, Gu N, Nah D, Hong K, Cho E, Sung K. Estimation of 24-hour urinary sodium excretion using spot urine samples. Nutrients 2014; 6: 2360-2375. PMID 24955740
60. Tanaka T, Okamura T, Miura K, Kadowaki T, Ueshima H, Nakagawa H, Hashimoto T. A simple method to estimate populational 24-h urinary sodium and potassium excretion using a casual urine specimen. J Hum Hypertens 2002; 16: 97-103. PMID 11850766 DOI 10.1038/sj.jhh.1001307

61. Tochikubo O, Sasaki O, Umemura S, Kaneko Y. Management of hypertension in high school students by using new salt titrator tape. Hypertension 1986; 8: 1164-1171. PMID 3793198
62. Toft U, Cerqueira C, Andreasen AH, Thuesen BH, Laurberg P, Ovesen L, Perrild H, Jorgensen T. Estimating salt intake in a caucasian population: Can spot urine substitute 24-hour urine samples? Eur J Prev Cardiol 2014; 21: 1300-1307. PMID 23559538
63. Trotter A, Stoll M, Leititis JU, Blatter A, Pohlandt F. Circadian variations of urinary electrolyte concentrations in preterm and term infants. J Pediatr 1996; 128: 253-256. PMID 8636824
64. Vanacor R, Soares R, Manica D, Furlanetto TW. Urinary iodine in 24 h is associated with natriuresis and is better reflected by an afternoon sample. Ann Nutr Metab 2008; 53: 43-49. PMID 18802329
65. Lee Watson R, Langford HG. Usefulness of Overnight Urines in Population Groups: Pilot Studies of Sodium, Potassium, and Calcium Excretion. Am J Clin Nutr 1970; 23: 290-304. PMID 5436638
66. Webster J, Su'a S, Ieremia M, Bompoint S, Johnson C, Faeamani G, Vaiaso M, Snowdon W, Land MA, Trieu K. Salt Intakes, Knowledge, and Behavior in Samoa: Monitoring Salt-Consumption Patterns Through the World Health Organization's Surveillance of Noncommunicable Disease Risk Factors (STEPS). J Clin Hypertens (Greenwich) 2016. PMID 26843490
67. Whiting SJ, Green TJ, MacKenzie EP, Weeks SJ. Effects of excess protein, sodium and potassium on acute and chronic urinary calcium excretion in young women. Nutrition Research 1998; 18: 475-487. DOI 10.1016/S0271-5317(98)00036-0
68. Wolf JP, Henriet MT, Nguyen NU, Dumoulin G, Laroze M, Berthelay S. Expression of plasma renin activity in terms of urinary sodium excretion and posture in normal subjects on free sodium intake. Ren Physiol 1984; 7: 237-42. PMID 6382488
69. Yamasue K, Tochikubo O, Kono E, Maeda H. Self-monitoring of home blood pressure with estimation of daily salt intake using a new electrical device. J Hum Hypertens 2006; 20: 593-598. PMID 16710288
70. Yamauchi T, Furuta M, Hamada J, Kondo T, Sakakibara H, Miyao M. Dietary salt intake and blood pressure among schoolchildren. Ann Physiol Anthropol 1994; 13: 329-336. PMID 7880322
71. Yamori Y, Kihara M, Fujikawa J, Soh Y, Nara Y, Ohtaka M, Horie R, Tsunematsu T, Note S, Kukase M. Dietary risk factors of stroke and hypertension in Japan -- Part 2: Validity of urinalysis for dietary salt and protein intakes under a field condition. Jpn Circ J 1982; 46: 939-43. PMID 7109210
