# Supplementary material for: Reliable Quantification of the Potential for Equations Based on Spot Urine Samples to Estimate Population Salt Intake: Protocol for a Systematic Review and Meta-Analysis
Source: JMIR Res Protoc. 2016 Sep 21;5(3):e190. doi: 10.2196/resprot.6282 (PMC5052460; doi:10.2196/resprot.6282)
Supplement: Multimedia Appendix 5 [file resprot_v5i3e190_app5.pdf]

MULTIMEDIA APPENDIX 5

TABLE: Identified studies for which full text unavailable and eligibility uncertain

**First author year**	**Title**
Allen NB 2014 [1]	The Validity of Predictive Equations for 24-Hour Urine Sodium Excretion Among Older Adults and Those with Hypertension: The MESA and CARDIA Urinary Sodium Study
Amarapurkar DN 1994 [2]	Role of routine estimation of creatinine clearance in patients with liver cirrhosis
Brandle E 1996 [3]	Can examination of spontaneous urine samples adequately replace 24-hour-urine samples for determining excretory rate of various lithogenic and inhibitory substances in metabolic evaluation of kidney calculi patients?
Brewster L 2010 [4]	Systematic review of the use of spot and overnight urine for assessment of sodium excretion
Bruce NG 1990 [5]	Casual urine concentrations of sodium, potassium, and creatinine in population studies of blood pressure
Campbell R 2009 [6]	Estimating sodium intake in the clinic: Use of spot urine measurements
Carbonell JM 1999 [7]	Urinary calcium and sodium excretion in healthy children
Cholongitas E 2012 [8]	Random spot urine sodium/potasium ratio is associated with renal dysfunction and outcome in patients with decompensated cirrhosis and ascites
Chouccair S 2015 [9]	Assessment of dietary sodium intake by spot urine and by a food frequency questionnaire: Validation against 24 hour urine collection
Choudhury S 2010 [10]	Daily salt intake estimated from urinary excretion of sodium in a Bangladeshi population
Cohall DH 2013 [11]	Predicting 24-hour urinary sodium excretion in afro-caribbean barbadians by comparing urine sodium excretion over different durations versus spot collection
Costa EA 1994 [12]	Diastolic pressure as an index of salt sensitivity
Damasio B 2005 [13]	Prevalence of fasting hypercalciuria associated with increased citraturia in the ambulatory evaluation of nephrolithiasis
Dishy V 2009 [14]	Rolofylline, an Adenosine A1 receptor antagonist increases urine output and sodium excretion in healthy subjects
Duzen O 2010 [15]	Steroid induced hypercalciuria; incidence, course and associated factors
Gerbes AL 1998 [16]	Renal effects of transjugular intrahepatic portosystemic shunt in cirrhosis: comparison of patients with ascites, with refractory

	ascites, or without ascites
Giampietro O<br>1992 [17]	Endogenous digitalis-like factors (EDLF) in obese individuals: preliminary results
Gisbertz C<br>2012 [18]	Renal denervation therapy in patients with resistant hypertension is associated by enhanced urinary sodium excretion
Greco M<br>2015 [19]	Comparison of three formulae to estimate 24-hour urinary sodium excretion using morning spot urine samples and correlation with conductivity
Haga M<br>2007 [20]	Estimation of overnight urine volume and 24-hour urine volume in healthy Japanese infants
Harm K<br>1988 [21]	24-hour excretion and morning urine concentration of clinical chemical urinary components in renal diseases
Hashimoto T<br>2007 [22]	The estimated salt intake by Kawasaki's method in response to a change in salt intake in patients with hypertension
Hashimoto T<br>2010 [23]	A large-scale survey of daily salt intake among outpatients in the Morioka area
Hashimoto T<br>2012 [24]	Casual urine method for estimating daily salt intake is influenced by the timing of urine collection and the actual daily salt intake in patients with hypertension
Holm EA<br>2009 [25]	Diagnosis of the syndrome of inappropriate secretion of antidiuretic hormone
Hsiao ZK<br>1986 [26]	Timed overnight sodium and potassium excretion and blood pressure in steel workers in North China
Kawano Y<br>2012 [27]	Self-measurement of daily salt intake from overnight urine using an electronic device in the management of hypertension
Kawasaki T<br>1986 [28]	Studies on estimation of 24-hour urinary sodium excretion from predicted creatinine excretion and fractional urine sodium/creatinine ratio
Kovacevic L<br>1998 [29]	Sodium excretion in children with lithogenic disorders
Krieg M<br>1986 [30]	Comparative quantitative clinico-chemical analysis of the characteristics of 24-hour urine and morning urine
Krieg M<br>1986 [31]	Quantitative analysis of clinical chemical parameters in the 24 h urine and in the morning urine: A comparative study
Kynast-Gales SA<br>1994 [32]	Effect of caffeine on circadian excretion of urinary calcium and magnesium
Lalau JD<br>1992 [33]	Vertebral density of hypercalciuric lithiasis. Its relation to calcium-protein intake and vitamin D metabolism
Leary WP<br>1984 [34]	The effects of various single doses of muzolimine on magnesiuria in healthy adults
Lee CC<br>2011 [35]	Diagnosis and treatment of hypokalemia
Luft FC<br>1980 [36]	Nocturnal urinary electrolyte excretion and its relationship to the renin system and sympathetic activity in normal and hypertensive man
Luft FC	The efficacy of quantitative and qualitative chloride titrators in

1985 [37]	the estimation of human salt intake
Luzardo L<br>2011 [38]	Clinical approach to sodium consumption
Mandai T<br>1985 [39]	Measurement of urinary sodium and potassium excretion with the proportional sampling device (Partition Cup) compared with the 24 hour whole urine collection
Maryam KJ<br>2012 [40]	Correlation between spot urine sodium, 24 hour urinary sodium and food frequency questionnaire in estimation of salt intake in healthy individuals
Matsushita K<br>1987 [41]	Significance of the calcium to creatinine concentration ratio of a single-voided urine specimen in patients with hypercalciuric urolithiasis
Miladipour AH<br>2011 [42]	Sodium intake and correlation of urine sodium in spot urine and 24-hour urine
Mill JG<br>2013	Validation study of random urine collection to estimate the daily intake of sodium and potassium
Minetti EE<br>1992 [43]	Urinary salt titrator stick: a useful and quick estimate of dietary sodium intake?
Mohii el SM<br>2013 [44]	Diagnostic usefulness of the random urine Na/K ratio in predicting therapeutic response for diuretics in cirrhotic patients with ascites
Moore M<br>1979 [45]	Spot urinary sodium/creatinine ratio predicts previous day's 24 hour sodium excretion in young essential hypertensives
Nerbass FB<br>2014 [46]	Development of a formula for estimation of sodium intake from spot urine in people with chronic kidney disease
Ohgitani S<br>1997 [47]	Correction of urinary calcium levels for urine osmotic pressure
Ohya Y<br>1994 [48]	Crossover comparison of the effects of enalapril and captopril on potassium homeostasis in patients with mild hypertension
Orejas RAG<br>1992 [49]	Type 1 diabetes mellitus and nephropathy. Predicting factors in pediatric age
Park IJ<br>1999 [50]	Diurnal Variation of urinary excretion of protein metabolites and electrolytes
Park JE<br>2010 [51]	Diagnostic usefulness of the random urine Na/K ratio in cirrhotic patients with ascites: a pilot study
Polonia J<br>1996 [52]	Reduction of the antihypertensive effects of enalapril by indomethacin. Its independence from renal sodium retention
Polonia J<br>1996 [53]	Attenuation by indomethacin of the antihypertensive effects of enalapril may be independent of sodium retention
Polonia J<br>2014 [54]	Estimation of 24-hour sodium, potassium and albumin excretion from spot urine samples in a national representative survey of hypertension (PHYSA)
Schulte AG<br>2002 [55]	Influence of fluoridated salt on urinary fluoride excretion of adults
Seric V<br>2009 [56]	Metabolic and physico-chemical urolithiasis parameters in the first morning urine

Servetto C 2014 [57]	Utility of the first morning urine in the evaluation of renal calculi risk factors
Subramanian S 2013 [58]	Spot urine tests in predicting 24-hour urine sodium excretion in Asian patients
Takase H 2013 [59]	Survey of 24-hour salt intake estimated by spot urine in Japanese general population; changes during 5 years
Tan TW 2011 [60]	Is 24-hour urinary metabolic evaluation in urolithiasis useful in the Singapore context?
Toft U 2012 [61]	Validation of a simple method to estimate populational 24-h urinary sodium excretion based on a casual urine specimen in an adult Danish population
Torffvit O 2004 [62]	Urinary excretion rate of Tamm-Horsfall protein is related to salt intake in humans
Tsai TJ 1991 [63]	Comparison between spot urine and overnight urine in the estimation of 24-hour excretion of urine protein, sodium and kallikrein
Tuomilehto J 1985 [64]	Value of overnight urines in the follow-up of people with borderline hypertensive trying to reduce their salt intake
Zanchetta JR 1991 [65]	Urinary excretion of sodium and magnesium in idiopathic hypercalciuria
Xi Y 2014 [66]	Research of the spot urine method in estimating 24-hour urinary sodium in hypertensive patients
[illegible] 2011	[illegible](The evaluation of estimating salt intake from spot urine)

REFERENCES

1. Allen NB, Zhao L, Loria C, Van Horn L, Wang CY, Pfieffer C, Cogswell M, Wright J, Liu K. The validity of predictive equations for 24-hour urine sodium excretion among older adults and those with hypertension: The MESA and CARDIA Urinary Sodium Study.

Circulation 2014; 129.
2. Amarapurkar DN, Dhawan P, Kalro RH. Role of routine estimation of creatinine clearance in patients with liver cirrhosis. Indian J Gastroenterol 1994; 13: 79-82. PMID 8076985
3. Brandle E, Melzer H, Gomez-Anson B, Flohr P, Kleinschmidt K, Sieberth HG, Hautmann RE. Can examination of spontaneous urine samples adequately replace 24-hour-urine samples for determining excretory rate of various lithogenic and inhibitory substances in metabolic evaluation of kidney calculi patients? Urologe A 1996; 35: 136-41. PMID 8650847
4. Brewster L, Bohte E, van Montfrans G. Systematic review of the use of spot and overnight urine for assessment of sodium excretion. J Hypertens 2010; 28: e230. DOI 10.1097/01.hjh.0000378892.59718.c0
5. Bruce NG, Cook DG, Shaper AG, Ratcliffe JG. Casual urine concentrations of sodium, potassium, and creatine in population studies of blood pressure. J Hum Hypertens 1990; 4: 597-602. PMID 2096199
6. Campbell RC, Aaron KJ, Krishna Reddy P. Estimating sodium intake in the clinic: Use of spot urine measurements. American Journal of Kidney Diseases 2009; 53 (4): A31. DOI 10.1053/j.ajkd.2009.01.055
7. Carbonell JM, Vazquez Martul M, Baeza J, Vila S, Arnaiz P, Ecija JL. Urinary calcium and sodium excretion in healthy children. [Spanish]. Nefrologia 1999; 19: 223-230.
8. Cholongitas E, Goulis J, Arsos G, Nakouti T, Kouvelis J, Birtsou C, Papadopoulou S, Karakatsanis K, Akriviadis E. Random spot urine sodium/potasium ratio is associated with renal dysfunction and outcome in patients with decompensated cirrhosis and ascites. Journal of Hepatology 2012; 56: S265. DOI 10.1016/S0168-8278(12)60682-5
9. Chouccair S, Naja F, Akl C, Helou R, Ismaeel H, Nasreddine L. Assessment of dietary sodium intake by spot urine and by a food frequency questionnaire: Validation against 24 hour urine collection. Obesity Facts 2015; 8: 70-71.
10. Choudhury S, Tabassum F, Ahmed J, Zaman M, Rouf A, Khandaker R, Malik A. Daily salt intake estimated from urinary excretion of sodium in a Bangladeshi population. Circulation 2010; 122 (2): e352-e353.
11. Cohall DH, Scantlebury-Manning T, Nakhleh C, Toure D, James S, Hall K. Predicting 24-hour urinary sodium excretion in Afro-Caribbean Barbadians by comparing urine sodium excretion over different durations versus spot collection. West Indian Med J 2013; 62: 181-185. PMID 24564036
12. Costa EA, Rose G, Klein C, Achutti A. Diastolic pressure as an index of salt sensitivity. J Hum Hypertens 1994; 8: 703-709. PMID 7807501
13. Damasio MB, Massarino F, Durand F, Banchero R, Bottino P, De Franchis V, Carmignani G, Cannella G. Prevalence of fasting hypercalciuria associated with increased citraturia in the ambulatory evaluation of nephrolithiasis. J Nephrol 2005; 18: 262-266. PMID 16013013
14. Dishy V, Mansoor G, Hwang E, Petry A, Bloomfield D, Lai E. Rolofylline, an Adenosine A1 receptor antagonist increases urine output and sodium excretion in healthy subjects. European Journal of Heart Failure, Supplement 2009; 8: ii64.
15. Duzen O, Erkoc R, Begenik H, Soyoral YU, Aldemir MN. Steroid induced hypercalciuria; incidence, course and associated factors. NDT Plus 2010; 3: iii311-iii312.
16. Gerbes AL, Gulberg V, Waggershauser T, Holl J, Reiser M. Renal effects of transjugular

intrahepatic portosystemic shunt in cirrhosis: Comparison of patients with ascites, with refractory ascites, or without ascites. Hepatology 1998; 28: 683-688. PMID 9731559
17. Giampietro O, Clerico A, Penno G, Gregori G, Del Chicca MG, Cionini R, Volpe L, Navalesi R. Endogenous digitalis-like factors (EDLF) in obese individuals: preliminary results. J Nucl Biol Med 1992; 36: 41-5. PMID 1333283
18. Gisbertz C, Fatum C, Franzen K, Leidinger A, Graf T, Himmel F, Bode F, Reppel M, Mortensen K, Schunkert H, Weil J. Renal denervation therapy in patients with resistant hypertension is associated by enhanced urinary sodium excretion. Circulation 2012; 126.
19. Greco M, Renis M, Bonerba MA, Sicuro F, Lobreglio G. Comparison of three formulae to estimate 24-hour urinary sodium excretion using morning spot urine samples and correlation with conductivity. Clinical Chemistry and Laboratory Medicine 2015; 53: S254.
20. Haga M, Sakata T. Estimation of overnight urine volume and 24-hour urine volume in healthy Japanese infants. [Japanese]. J Nutr Sci Vitaminol (Tokyo) 2007; 60: 213-220. PMID 10.4327/jsnfs.60.213
21. Harm K, Zeiser T. 24-hour excretion and morning urine concentration of clinical chemical urinary components in renal diseases. [German]. Arztliche Laboratorium 1988; 34: 111-117.
22. Hashimoto T, Owada M, Sugawara T, Kawamura M. The estimated salt intake by Kawasaki's method in response to a change in salt intake in patients with hypertension. J Blood Pressure 2007; 14: 83-84.
23. Hashimoto T, Owada M, Kawamura M, Ogino T. A large-scale survey of daily salt intake among outpatients in the Morioka area. Med J Iwate Pref Hosp 2010; 49: 86-91.
24. Hashimoto T, Yagami F, Hashimoto Y, Sakamoto K, Owada M, Sugawara T, Kawamura M. Casual urine method for estimating daily salt intake is influenced by the timing of urine collection and the actual daily salt intake in patients with hypertension. J Hypertens 2012; 30: e304. DOI 10.1097/01.hjh.0000420504.07316.88
25. Holm EA, Bie P, Ottesen M, Odum L, Jespersen B. Diagnosis of the syndrome of inappropriate secretion of antidiuretic hormone. South Med J 2009; 102: 380-384. PMID 19279540
26. Hsiao Z, Wang S, Hong ZG, Liu K, Cheng T, Stamler J, Tao S. Tinned Overnight Sodium and Potassium Excretion and Blood Pressure in Steel Workers in North China. J Hypertens 1986; 4: 345-350. PMID 3734452
27. Kawano Y, Iwashima Y, Hayashi S, Yoshihara F, Nakamura S. Self-measurement of daily salt intake from overnight urine using an electronic device in the management of hypertension. J Hypertens 2012; 30: e305-e306. PMID 10.1097/01.hjh.0000420509.86230.ba
28. Kawasaki T, Uezono K, Utsunomiya H, Imamura K, Kikkawa K, Ueno M, Fujishima M. Studies on estimation of 24-hour urinary sodium excretion from predicted creatinine excretion and fractional urine sodium/creatinine ratio. J Health Sci 1986; 8: 57-63.
29. Kovacevic L, Kovacevic S, Smoljanic Z, Peco-Antic A, Kostic N, Gajic M, Kovacevic N, Jovanovic O. Sodium excretion in children with lithogenic disorders. [Croatian]. Srp Arh Celok Lek 1998; 126: 321-326. PMID 9863402
30. Krieg M, Gunsser K, Steinhagen-Thiessen E, Becker H. Comparative quantitative

clinico-chemical analysis of the characteristics of 24-hour urine and morning urine. J Clin Chem Clin Biochem 1986; 24: 863-869. PMID 3806015
31. Krieg M, Gunsser KJ, Steinhagen-Thiessen E, Becker H. Quantitative analysis of clinical chemical parameters in the 24 h urine and in the morning urine: A comparative study. [German]. Journal of Clinical Chemistry and Clinical Biochemistry 1986; 24: 863-869. PMID 3806015
32. Kynast-Gales SA, Massey LK. Effect of caffeine on circadian excretion of urinary calcium and magnesium. J Am Coll Nutr 1994; 13: 467-472. PMID 7836625
33. Lalau JD, Achard JM, Bataille P, Bergot C, Jans I, Boudailliez B, Petit J, Henon G, Westeel PF, el Esper N, et al. Vertebral density of hypercalciuric lithiasis. Its relation to calcium-protein intake and vitamin D metabolism. Ann Med Interne (Paris) 1992; 143: 293-8. PMID 1482031
34. Leary WP, Reyes AJ, Van der Byl K. The effects of various single doses of muzolimine on magnesiuria in healthy adults. Current Therapeutic Research - Clinical and Experimental 1984; 36: 163-170.
35. Lee CC, Huang WT, Lin SH. Diagnosis and treatment of hypokalemia. [Chinese]. Journal of Internal Medicine of Taiwan 2011; 22: 31-39.
36. Luft FC, Weinberger MH, Grim CE. Nocturnal urinary electrolyte excretion and its relationship to the renin system and sympathetic activity in normal and hypertensive man. J Lab Clin Med 1980; 95: 395-406. PMID 6986446
37. Luft F, Aronoff G, Sloan R, Fineberg N, Miller J, Free A. The efficacy of quantitative and qualitative chloride titrators in the estimation of human salt intake. Klin Wochenschr 1985; 63: 62-67. PMID 3981952
38. Luzardo L, Sottolano M, Lujambio I, Boggia J, Barindelli A, Noboa O. Clinical approach to sodium consumption. [Spanish]. Revista Medica del Uruguay 2011; 27: 228-235.
39. Mandai T, Ozawa H, Yano A. Measurement of urinary sodium and potassium excretion with the proportional sampling device (Partition Cup) compared with the 24 hour whole urine collection. [Japanese]. Japanese Journal of Nephrology 1985; 27: 815-823. PMID 4079114
40. Maryam KJ, Nani N, Rahman AR. Correlation between spot urine sodium, 24 hour urinary sodium and food frequency questionaire in estimation of salt intake in healthy individuals. J Hypertens 2012; 30: e305. DOI 10.1097/01.hjh.0000420507.92068.00
41. Matsushita K, tanikawa K. Significance of the calcium to creatinine concentration ratio of a single-voided urine specimen in patients with hypercalciuric urolithiasis. Tokai J Exp Clin Med 1987; 12: 167-171. PMID 3454077
42. Miladipour AH, Parvin M. Sodium intake and correlation of urine sodium in spot urine and 24-hour urine. Iranian Journal of Kidney Diseases 2011; 5: 50-51.
43. Minetti E, Airaghi C, Cozzi M, Guidi E. Urinary salt titrator stick: a useful and quick estimate of dietary sodium intake? J Hum Hypertens 1992; 6: 287-289. PMID 1433164
44. Mohii SM, El Mansy IM, Salah M, Khedr MA. Diagnostic usefulness of the random urine Na/K ratio in predicting therapeutic response for diuretics in cirrhotic patients with ascites. J Egypt Soc Parasitol 2013; 43: 767-776. PMID 24640876
45. Moore M, Burgess R, Volosin K, Buckalew V. Spot urinary sodium-creatinine ratio predicts previous days 24 hour sodium excretion in young essential hypertensives. Preventive Medicine 1979; 8: 200-200.

46. Nerbass FB, Pecoits-Filho R, McIntyre NJ, McIntyre CW, Taal MW. Development of a formula for estimation of sodium intake from spot urine in people with chronic kidney disease. Nephron Clin Pract 2014; 128: 61-66. PMID 25342580
47. Ohgitani S, Fujii Y, Fujita T. Correction of urinary calcium levels for urine osmotic pressure. Japanese Journal of Geriatrics 1997; 34: 409-414. PMID 9267153
48. Ohya Y, Ueno M, Takata Y, Tominaga M, Ohmori S, Kobayashi K, Abe I, Fujishima M. Crossover comparison of the effects of enalapril and captopril on potassium homeostasis in patients with mild hypertension. Int J Clin Pharmacol Ther 1994; 32: 655-9. PMID 7881704
49. Orejas RAG, del Molino Anta A, Santos Rodriguez F, Crespo Hernandez M, Garcia Vicente S, Rey Galan C, Macho Montoya V, Malaga Guerrero S. Type 1 diabetes mellitus and nephropathy. Predicting factors in pediatric age. An Esp Pediatr 1992; 37: 37-41. PMID 1416521
50. Park IJ, Jeon HS, Kwak YS, Shin OH. Diurnal variation of urinary excretion of protein metabolites and electrolytes. Korean Journal of Clinical Pathology 1999; 19: 404-408.
51. Park JE, Lee CH, Kim BS, Shin IH. Diagnostic usefulness of the random urine Na/K ratio in cirrhotic patients with ascites: a pilot study. Korean J Hepatol 2010; 16: 66-74. PMID 20375644
52. Polonia J, Gama G, Santos A. Reduction of the antihypertensive effects of enalapril by indomethacin. Its independence from renal sodium retention. [Portuguese]. Rev Port Cardiol 1996; 15: 485.
53. Polonia J, Gama G, Santos A. Attenuation by indomethacin of the antihypertensive effects of enalapril may be independent of sodium retention. [Portuguese]. Rev Port Cardiol 1996; 15: 485-492+460. PMID 8755685
54. Polonia J, Martins L, Pinto F, Nazare J. Estimation of 24-hour sodium, potassium and albumin excretion from spot urine samples in a national representative survey of hypertension (PHYSA). Journal of the American Society of Hypertension 2014; 1): e83. DOI 10.1016/j.jash.2014.03.194
55. Schulte AG, Graber R, Kasperk C, Koch MJ, Staehle HJ. Influence of fluoridated salt on urinary fluoride excretion of adults. Caries Res 2002; 36: 391-397. PMID 12459610
56. Seric V, Dutour-Sikiric M, Mihaljevic I, Tucak-Zoric S, Bilic-Curcic I, Babic-Ivancic V. Metabolic and physico-chemical urolithiasis parameters in the first morning urine. Coll Antropol 2009; 33 Suppl 2: 85-92. PMID 20120404
57. Servetto C, Bidegain E, Varela B. Utility of the first morning urine in the evaluation of renal calculi risk factors. Clinical Chemistry and Laboratory Medicine 2014; 52: S1084.
58. Srinivas S, Teo B, Toh Q, Koh Y, Li J, Sethi S, Lee EJC. Spot urine tests in predicting 24-hour urine sodium excretion in Asian patients. J Ren Nutr 2013; 23: 450-455. PMID 23402913
59. Takase H, Okado T, Tanaka T, Hashimoto T, Toriyama T, Hayashi H, Dohi Y. Survey of 24-hour salt intake estimated by spot urine in Japanese general population; changes during 5 years. Eur Heart J 2013; 34: 470.
60. Tan TW, Png KS, Lee YM, Chia SJ, Chong YL. Is 24-hour urinary metabolic evaluation in urolithiasis useful in the Singapore context? Annals of the Academy of Medicine Singapore 2011; 1): S88.
61. Toft U, Cerquira C, Jorgensen T. Validation of a simple method to estimate populational 24-h urinary sodium excretion based on a casual urine specimen in an adult danish population. Circulation 2012; 125.

62. Torffvit O, Melander O, Hulten UL. Urinary excretion rate of Tamm-Horsfall protein is related to salt intake in humans. Nephron Physiol 2004; 97: 31. PMID 15153749
63. Tsai TJ, Chen YM, Hsieh BS, Chen WY. Comparison between spot urine and overnight urine in the estimation of 24-hour excretion of urine protein, sodium and kallikrein. J Formos Med Assoc 1991; 90: 755-759. PMID 1683368
64. Tuomilehto J, Tanskanen A, Pietinen P. Value of overnight urines in the follow-up of people with borderline hypertensive trying to reduce their salt intake. Acta Cardiol 1985; 40: 325-328. PMID 3927646
65. Zanchetta JR, Bogado CE. Urinary excretion of sodium and magnesium in idiopathic hypercalciuria. Medicina (B Aires) 1991; 51: 296-302. PMID 1821917
66. Xi Y, Sun N, Zhao L, Wang H, Chen Y, Liu X, Han W. Research of the spot urine method in estimating 24-hour urinary sodium in hypertensive patients. Cardiovascular Research 2014; 103: S46. DOI 10.1093/cvr/cvu082.187
